# *Brassicaceae* microgreens: A novel and promissory source of sustainable bioactive compounds

**DOI:** 10.1016/j.crfs.2023.100480

**Published:** 2023-03-10

**Authors:** Florencia P. Alloggia, Roberto F. Bafumo, Daniela A. Ramirez, Marcos A. Maza, Alejandra B. Camargo

**Affiliations:** a Laboratorio de Cromatografía para Agroalimentos, Instituto de Biología Agrícola de Mendoza, CONICET y Facultad de Ciencias Agrarias, Universidad Nacional de Cuyo, Alte. Brown 500, Chacras de Coria, Mendoza, Argentina; b Cátedra de Química Analítica, Facultad de Ciencias Agrarias, UNCuyo, Mendoza, Argentina Institución, Alte. Brown 500, Chacras de Coria, Mendoza, Argentina; c Cátedra de Enología I, Facultad de Ciencias Agrarias, UNCuyo, Mendoza, Argentina Institución, Alte. Brown 500, Chacras de Coria, Mendoza, Argentina

**Keywords:** *Brassicaceae* microgreens, Agricultural practices, Bioactive compounds, Glucosinolates, Isothiocyanates, Phenolic compounds

## Abstract

Microgreens are novel foods with high concentrations of bioactive compounds and can be grown easily and sustainably. Among all the microgreens genera produced, *Brassicaceae* stand out because of the wide evidence about their beneficial effects on human health attributed to phenolic compounds, vitamins, and particularly glucosinolates and their breakdown products, isothiocyanates and indoles. The phytochemical profile of each species is affected by the growing conditions in a different manner. The agronomic practices that involve these factors can be used as tools to modulate and enhance the concentration of certain compounds of interest. In this sense, the present review summarizes the impact of substrates, artificial lighting, and fertilization on bioactive compound profiles among species. Since *Brassicaceae* microgreens, rich in bioactive compounds, can be considered functional foods, we also included a discussion about the health benefits associated with microgreens’ consumption reported in the literature, as well as their bioaccessibility and human absorption. Therefore, the present review aimed to analyze and systematize cultivation conditions of microgreens, in terms of their effects on phytochemical profiles, to provide possible strategies to enhance the functionality and health benefits of *Brassicaceae* microgreens.

## Highlights

• *Brassicaceae* microgreens present high concentrations of bioactive compounds.
• Phytochemical profiles of Brassicaceae microgreens are affected by growing conditions.
• Agronomic practices that enhance the concentration of bioactive compounds of interest.
• Functionality attributed to phenolic compounds, vitamins and glucosinolates.
• Beneficial effects on human health related to microgreens intake.

## Graphical abstract

**Figure:**
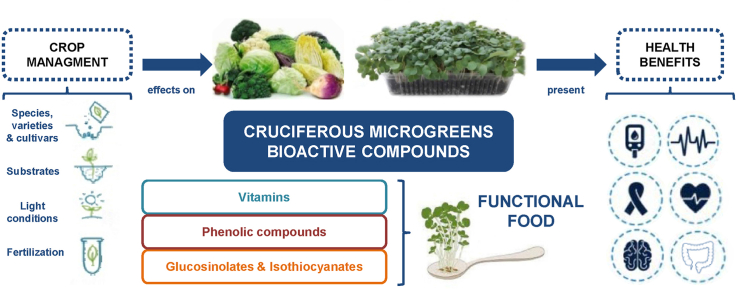

## 1 Introduction

Microgreens have gained increasing attention in the last few years as a novelty food. The consumption of these edible seedlings with fully developed cotyledons, and the hint of the first true leaves (Verlinden, 2020) have become very popular. This fact is a result of healthier eating trends focusing on functional foods with high content of phytochemicals, vitamins, and minerals (Kyriacou et al., 2016). In addition, microgreens are obtained sustainably and have the versatility to adapt to different cropping systems. It is possible to grow them both on large-scale greenhouses with soilless or hydroponic systems, as on a smaller scale such as home production for self-consumption (Renna et al., 2017). Therefore, these characteristics, added to its striking sensory attributes, promote great motivation for their research.

Regarding production, certain advantages should be highlighted. First, is their short growth cycle, which is around 7 and 21 days depending on the species. Furthermore, it is possible to use soils and various other substrates for soilless cultivation (Verlinden, 2020), large areas are not required, and the crop adapts very well to controlled indoor growing systems. Certain cultural practices, like fertilization, can be omitted and other practices, such as phytosanitary treatments or weed control, are not carried out. However, the production of microgreens also presents challenges to resolve as high seed requirements, moderate yields, and short shelf life (Kyriacou et al., 2016; Zhang et al., 2021).

Species and varieties selection is a fundamental factor in microgreens production. Ebert, detailed some commonly used crop groups for microgreens production, such as legumes, cereals, pseudocereals, oilseeds, vegetables, and herbs (Ebert, 2022). In this sense, Di Gioia et al. also discussed the importance of assessing the edibility of species at the seedling stage as in the case of the potential use of wild species (Gioia et al., 2017). Among the most widespread crops, species and varieties from the *Brassicaceae* family are widely used (Ebert, 2022). Consumption of vegetables from this family is recommended for their phytochemical richness (Fusari et al., 2020), and related functional properties. Most of the bioactive compounds are products of secondary metabolism; the main ones are glucosinolates and their breakdown products, isothiocyanates and indoles. In addition, phenolic compounds, carotenoids, tocopherols, and ascorbic acid are compounds of great interest in this family (Ramirez et al., 2020). Several biological activities are associated with these compounds being the cancer-protective effects of glucosinolates and isothiocyanates the most studied among others such as antioxidant, anti-inflammatory, anti-diabetic, neuroprotective, and cholesterol-lowering effects of *Brassicaceae* vegetables (Ramirez et al., 2020; Ebert, 2022). The noteworthy fact in microgreens, is that numerous studies report higher concentrations of the bioactive compounds mentioned above, compared to their mature counterparts, making them a healthy eating alternative (Kyriacou et al., 2016). In this sense, given that the biosynthesis of several secondary plant metabolites is triggered by environmental and agronomical stressing factors, the management of them could become a mechanism for optimize profiles and concentrations of bioactive compounds (Neugart et al., 2018; Ebert, 2022). Based on this approach, the present review aims to resume the main findings of the last 10 years regarding growing conditions of *Brassicaceae* microgreens, and their effect on bioactive compound concentrations to provide recommendations for crop management practices that enhance the functional quality of microgreens.

The search for this review was carried out using the keywords Microgreens - Brassicaceae - Seed - Fertigation - Light - Substrate in digital repositories such as Scopus and Scholar Google. An exclusion criterion was applied according to the date of publication, including studies published between 2010 and 2022. This is the first time that microgreens' cultivation conditions have been summarized and discussed in terms of their effect on their phytochemical profiles providing insights into possible strategies to enhance functionality and health benefits.

## 2 Crop management

### 2.1 Species, varieties, and cultivars

It is possible to find edible microgreens from different plant genres. We focus on those belonging to the *Brassicaceae* family, as previously stated, because of their rich functionality.

This botanical family includes 360 genera (Paterson et al., 2000), with several species of economic importance as horticultural crops (Li et al., 2018) of worldwide distribution (Ramirez et al., 2020), and also occupies an important place among commonly cultivated crops as microgreens (Ebert, 2022). The choice of a particular genotype becomes important since each of them has a characteristic phytochemical profile (Kyriacou et al., 2021a, Kyriacou et al., 2021b). However, variations in the phytochemical levels reported could be explained not only by genotypes but also the growing conditions (Johnson et al., 2021), the growth stage considered (Choe et al., 2018; Ebert, 2022) and even by the methods of detection and extraction of metabolites employed.

**Fig. 1:**
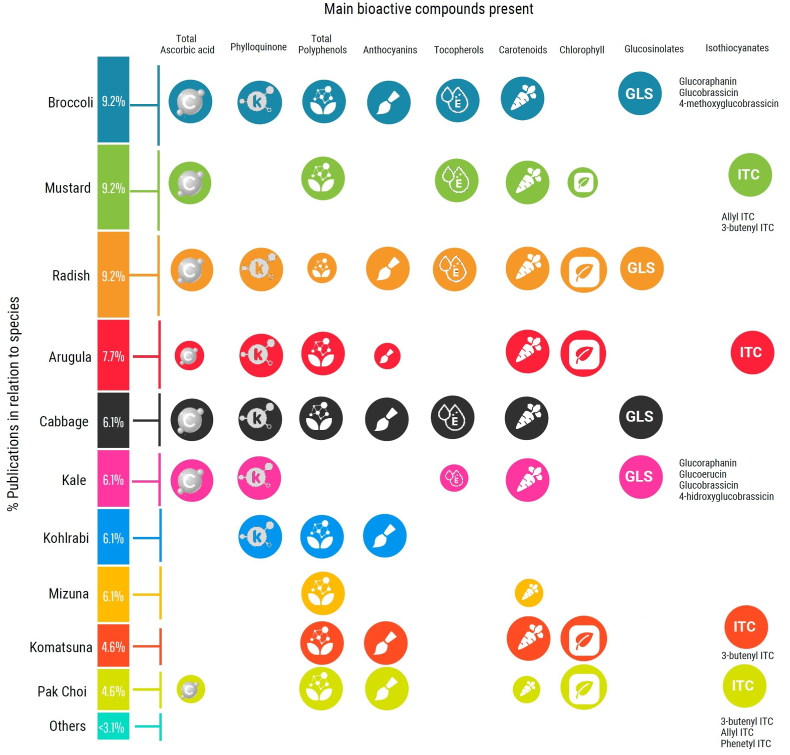
In the colored column are represented the percentages of each specie resulting from the bibliographic search. The icons on the right represent the different bioactive compounds, and the circle sizes are related to the amounts found.

Table 1–a resumes the bibliography searched, and the obtained results are plotted in Fig. 1. There, it is possible to observe on the first column the species ordered from highest to lowest according to the number of publications of each one. Broccoli, mustard and radish are the main studied species, followed by arugula, cabbage, kale, kohlrabi, and mizuna. The most studied species are also the most widespread commercial microgreens, which could be explained both by their acceptability and the availability of seeds. In addition, it should be noted that the main studied species, such as broccoli, radish and cabbage, have the highest diversity of bioactive compounds reported. These include vitamins as well as phenolic compounds and glucosinolates. On the other hand, among the less studied species, the diversity of reported bioactive compounds is lower. Some of these species have started to become more widespread in recent years, and further studies are needed to evaluate their phytochemical profiles in depth. Among this group we can highlight cauliflower, which turned out to be an important source of vitamin C, or cress as a source of vitamin C, vitamin A, and glucosinolates.

**Table 1-a:** Species and varieties of *Brassicaceae* microgreens that were studied for their phytochemical profile.

Common name	Type	Species/Cultivars	References
Broccoli		*Brassica oleracea* L. *var. italica*	Kowitcharoen et al. (2021)
			Johnson et al. (2021)
			Marchioni et al. (2021)
			Xiao et al. (2019a)
		*Brassica oleracea* L. *Group italica Plenk* var. *‘Mugnuli’*	Paradiso et al. (2018)
		*Brassica oleracea* L. *Group italica Plenk cv. ‘Natalino’*	
		*Brassica oleracea* var. *italica ‘Broccolo Nero’*	Di Bella et al. (2021)
		*Brassica oleracea* var. *italica ‘Cavolo Broccolo Ramoso Calabrese’*	
Brussels sprouts		*Brassica oleracea* L. *var. gemmifera*	Xiao et al. (2019a)
Cabbage	Red	*Brassica oleracea* L. *var. capitata f. rubra*	Sun et al. (2013)
			Xiao et al. (2019a)
			Johnson et al. (2021)
			Kowitcharoen et al. (2021)
	Chinese	*Brassica rapa* L. *var. pekinensis*	Xiao et al. (2019a)
	Green	*Brassica oleracea* L. *var. capitata f. alba*	Xiao et al. (2019a)
	Savoy	*Brassica oleracea* L. *var. capitata f. sabauda*	Xiao et al. (2019a)
Cauliflower		*Brassica oleracea* L. *var. botrytis*	Xiao et al. (2019a)
Collard		*Brassica oleracea* L. *var. viridis*	Xiao et al. (2019a)
Cress		*Lepidium sativum* L.	Kyriacou et al. (2019)
Daikon		*Raphanus raphanistrum* subsp. *sativus L. Domin*	Marchioni et al. (2021)
Kale		*Brassica oleracea* var. *acephala*	Di Bella et al. (2021)
		*DC. ‘Cavolo Laciniato Nero di Toscana’*	
		*Brassica oleracea* L.	Wojdyło et al. (2020)
	Chinese	*Brassica oleracea* L. *var. alboglabra*	Kowitcharoen et al. (2021)
			Xiao et al. (2019a)
	Red	*Brassica oleracea* L. *var. acephala*	Xiao et al. (2019a)
	Tucsan	*Brassica oleracea* L. *var. acephala*	Xiao et al. (2019a)
Kohlrabi	Purple	*Brassica oleracea* L. *var*.*gongylodes*	Kyriacou et al. (2019)
			Xiao et al. (2012)
			Sun et al. (2013)
			Xiao et al. (2019a)
Komatsuna		*Brassica rapa* L. *var. perviridis*	Kyriacou et al. (2019)
			Kyriacou et al. (2021)
			Xiao et al. (2019a)
Mibuna		*Brassica rapa* L. *subsp. nipposinica*	Kyriacou et al. (2019)
			Kyriacou et al. (2021)
Mizuna		*Brassica rapa* L. *var. nipposinica*	Sun et al. (2013)
		*Brassica rapa* L. *var. nipposinica*	Xiao et al. (2012)
			Xiao et al. (2019a)
		*Brassica rapa* L. *var. japonica cv. Greens*	Kyriacou et al. (2021)
Mustard		*Brassica juncea* L. *Czern.*	(Ghoora et al., 2020)
			Kyriacou et al. (2019)
			Marchioni et al. (2021)
	Purple	*Brassica juncea* L. *Czern.*	Sun et al. (2013)
			Xiao et al. (2012)
	Red	*Brassica juncea* L. *Czern.*	Sun et al. (2013)
			Xiao et al. (2012)
			Xiao et al. (2019a)
	Dijon	*Brassica juncea* L. *Czern.*	Xiao et al. (2019a)
Pakchoi		*Brassica rapa* L. *var. chinensis*	Kyriacou et al. (2019)
			Kyriacou et al. (2021)
			Xiao et al. (2019a)
Peppercress		*Lepidium bonariense* L.	Xiao et al. (2012)
			Xiao et al. (2019a)
Radish	China rose	*Raphanus sativus* L.	Xiao et al. (2012)
			Xiao et al. (2019a)
	Green daikon	*Raphanus sativus* L*.var. longipinnatus*	Xiao et al. (2012)
		*Raphanus sativus* L. *var. Imp. Chetki*	(Ghoora et al., 2020)
	Red	*Raphanus sativus* L.	Xiao et al. (2019a)
	Ruby	*Raphanus sativus* L.	Xiao et al. (2019a)
	Rat-tailed	*Raphanus caudatus* L. *var. caudatus Alef.*	Kowitcharoen et al. (2021)
	Daikon	*Raphanus sativus* L. *var*. *longipinnatus*	Xiao et al. (2019a)
	Opal	*Raphanus sativus* L.	Xiao et al. (2012)
	Purple	*Raphanus sativus* L. *var. longipinnatus*	Kowitcharoen et al. (2021)
		*Raphanus sativus* L.	Kowitcharoen et al. (2021)
			Kyriacou et al. (2019)
			Wojdyło et al. (2020)
Rapini		*Brassica rapa* L. *var. ruvo*	Xiao et al. (2019a)
Rocket/Arugula		*Eruca vesicaria* L. *Cav.*	Marchioni et al. (2021)
		*Eruca sativa Mill.*	(Baldi et al., 2015)
			Xiao et al. (2012)
			Xiao et al. (2019a)
			Johnson et al. (2021)
Rutabaga		*Brassica napus* L. *var. napobrassica*	Xiao et al. (2019a)
Tatsoi		*Brassica rapa* L. *subsp. narinosa*	Kyriacou et al. (2019)
		*Brassica narinosa* L. *var. rosularis*	Xiao et al. (2019a)
Turnip		*Brassica rapa* L. *var. rapa*	Xiao et al. (2019a)
Upland cress		*Barbarea verna P. Mill. Aschers*	Xiao et al. (2019a)
Wall rocket		*Diplotaxis erucoide*	Guijarro-Real et al. (2020)
Wasabi		*Wasabia japonica Matsum.*	Xiao et al. (2012)
			Xiao et al. (2019a)
Watercress		*Nasturtium officinale R.Br.*	Marchioni et al. (2021)
			Xiao et al. (2019a)

Regarding phytochemical profiles, the contents of total ascorbic acid, phylloquinone, total polyphenols, anthocyanins, tocopherols, carotenoids, chlorophyll, glucosinolates, and isothiocyanates are generally analyzed. Although organosulfur compounds are the hallmark bioactive compounds of Brassicas, they are not the most frequently reported compounds. Table 1–b summarizes a detailed description of bioactive compounds reported at high levels in Brassicaceae microgreens.

**Table 1-b:** Detailed description of bioactive compounds in Brassicaceae microgreens.

Bioactive compounds	Brassicaceae microgreens species with high contents and major compounds identified∗
Phenolic compounds	Broccoli; red cabbage; cress (quercetin-3-O-glucoside and kaempferol-3-O-glucoside); daikon; kohlrabi (kaempferol-3-O-(caffeoyl)-sophoroside-7- O-glucoside); mibuna (quercetin-3-O-(feruloyl)-sophoroside-7-O-glucoside and isorhamnetin-3-gentiobioside); pakchoi (quercetin-3-O-(feruloyl)-sophoroside-7-O-glucoside and kaempferol-3-O-(caffeoyl)-sophoroside-7- O-glucoside); and tatsoi (quercetin-3-O-(feruloyl)-sophoroside-7-O-glucoside and kaempferol-3-O-(caffeoyl)-sophoroside-7- O-glucoside)
Anthocyanins	Broccoli; purple radish; red cabbage (cyanidin 3-diferuloyl-sophoroside- 5-glucoside, cyanidin 3 (sinapoyl) (sinapoyl)sophoroside-5-glucoside, and cyanidin 3-(sinapoyl) (feruloyl)sophoroside- 5-glucoside); kohlrabi((feruloyl) (feruloyl), (sinapoyl) (feruloyl) and (sinapoyl) (sinapoyl) cyanidin 3-diglucoside-5-(malonyl)-glucoside); komatsuna (cyanidin-3-(feruloyl)(sinapoyl)dihexoside-5-hexoside); and pakchoi (cyanidin-3-(feruloyl)(sinapoyl)dihexoside-5-hexoside)
Glucosinolates	Broccoli (glucoraphanin, glucobrassicin, and 4-methoxyglucobrassicin); kale (glucoraphanin and glucobrassicin); chinese cabbage; cress; komatsuna; radish; and tatsoi
Isothiocyanates and indoles	Komatsuna (3-butenyl isothiocyanate); mibuna (3-butenyl isothiocyanate, allyl isothiocyanate, and phenetyl isothiocyanate); pakchoi (3-butenyl isothiocyanate, allyl isothiocyanate, and phenetyl isothiocyanate); mustard (allyl and 3-butenyl isothiocyanate); and wall rocket (allyl isothiocyanate)
Vitamin A	Cauliflower, broccoli, Brussels sprouts, upland cress, watercress, tuscan kale, chinese kale, mibuna, mustard, tatsoi, and wasabi
Vitamin C	Cauliflower, broccoli, Brussels sprouts, cress, Chinese kale, mustard, and radish
Vitamin E	Radish
Vitamin K	Broccoli, Brussels sprouts, tuscan kale, daikon, and turnip

∗ Major compounds are detailed in brackets.

About phenolic compounds, authors mostly quantify total content. Several studies have also established the qualitative and quantitative phenolic profile. Species-rich in total polyphenols are broccoli, red cabbage, cress, daikon, kohlrabi, mibuna, pakchoi and tatsoi, with contents of total polyphenols in a range between 774 and 2645 mg kg-1 dw. Major phenolic compounds identified by specie are detailed in Table 1–b. Flavonols glycosides are the main phenolic compounds present in Brassica microgreens, with predomination of kaempferol, quercetin and isorhamnetin glycosides (Kyriacou et al., 2019). Although this is a deep-studied group of compounds in mature vegetables of Brassicaceae family, the data about microgreens’ phenolic compounds is scarce. Given the importance for human health of this group of compounds’ consumption due to their well known antioxidant activity, it is of interest to further study microgreens as a possible source of phenolic compounds.

Regarding anthocyanins, the highest contents were detected in purple leaf species, as expected (Kyriacou et al., 2021). In root species, such as radish, the richest variety in anthocyanins was also the purple radish (Wojdyło et al., 2020; Kowitcharoen et al., 2021). In contrast, low levels were reported in arugula by Marchioni (Marchioni et al., 2021). Among the species with high anthocyanins content presented in Table 1–b, we can mention broccoli, purple radish, red cabbage, kohlrabi, komatsuna, and pakchoi (Sun et al., 2013; Marchioni et al., 2021). When identifying anthocyanins components Kyriacou et at (Kyriacou et al., 2021). agreed that cyanidin-3-(feruloyl)(sinapoyl)dihexoside-5-hexoside was the most abundant, despite the species. Knowledge of anthocyanin content is useful when starting a cultivation to select purple leaf species considering their functional value as well as their visual attractiveness.

In addition, within the group of vitamins and their precursors, various compounds are usually analyzed. From the reported data, it is noteworthy that cauliflower is a rich source of vitamin C (Xiao et al., 2019a). Likewise, we can mention broccoli (Xiao et al., 2019a; Kowitcharoen et al., 2021), Brussels sprouts (Xiao et al., 2019a), cress (Kyriacou et al., 2019), Chinese kale (Xiao et al., 2019a; Kowitcharoen et al., 2021), mustard (Marchioni et al., 2021) and radish (Ghoora et al., 2020) among the species with important levels of ascorbic acid. Reported contents in microgreens range from 89.3 mg/100 g FW to 18.9 mg/100 g FW. In terms of vitamin K content, broccoli, Brussels sprouts, tuscan kale, daikon and turnip microgreens are characterized by their high level of phylloquinone. Authors highlight the higher levels of phylloquinone found in microgreens compared to their mature counterparts, especially in edible roots species (Xiao et al., 2019a). High contents of vitamin A are reported in rapini, tatsoi, upland cress and watercress (Xiao et al., 2019a), tuscan and chinese kale (Kowitcharoen*et al.*, 2021), mibuna (Kyriacou et al., 2021), and mustard (Ghoora et al., 2020). These microgreen species are considered rich sources of vitamin A as their total carotenoid levels exceed 10 mg/100 g of edible portion (Xiao et al., 2012, 2019b). Finally, about vitamin E, several articles agree radish is a rich source of α-tocopherol. In this regard, we note that the range of levels reported for radish microgreens presents a wide variation among different studies, from 4.1 to 58.6 mg/100 g FW. Therefore, in order to analyze and compare experiments with different conditions, it would be convenient to compare the relative levels between species (Xiao et al., 2012; 2019a; Ghoora et al., 2020).

Regarding *Brassicaceae* microgreens specific bioactive compounds, authors generally are focused on glucosinolates content. In this sense broccoli, kale (Di Bella et al., 2021), chinese cabbage, cress, komatsuna, radish and tatsoi (Xiao et al., 2019a) showed high levels. Contradictory results were found for arugula, while Johnson et al. reported uniquely high levels (Johnson et al., 2021), Xiao et al. indicated arugula did not stand out by its glucosinolates levels (Xiao et al., 2019a). On the other hand, among the species with low content of glucosinolates they can be mentioned cauliflower, mustard and wasabi (Xiao et al., 2019a). Among the studies that performed a glucosinolates profiling, Di Bella et al. reported glucoraphanin, glucobrassicin, and 4-methoxyglucobrassicin in broccoli; and glucoraphanin and glucobrassicin in kale (Di Bella et al., 2021). They emphasize in this work that the glucosinolates profile is influenced not only by genotype but also by climatic conditions and growth stage. However, the influence of microgreens growing conditions on these compounds and their degradation products, still needs to be further investigated in depth. In this regard, it is worth focusing on isothiocyanates and indoles, the degradation products of glucosinolates, as they are the ones that exert biological activity. In this sense, Kyriacou et al. (Kyriacou et al., 2021) determined particular isothiocyanates in komatsuna, mibuna, and pakchoi microgreens (Table 1–b). Furthermore, other researchers have reported isothiocyanate profiles in mustard (Marchioni et al., 2021) and wall rocket (Guijarro-Real et al., 2020). The relative abundance of these degradation products is generally related to the glucosinolates of origin. Isothiocyanates derived from aromatic and aliphatic glucosinolates are usually the majority. Nonetheless, the information in this regard is still incipient and it is necessary to deepen and extend the research in this line to other species.

### 2.2 Substrates

Substrates provide crops not only support and anchor but also store water, oxygen and nutrients (Puustjärvi, 1974). In this sense, soil is the natural substrate that meets these conditions. However, with the development of agriculture, diverse alternative materials to use as substrate arose.

Microgreens can be grown in different systems, from home production (Ebert, 2022) to plant factories (Bantis, 2021) employing hydroponic or soilless cultivation (Nolan, 2018). For that reason, it is possible to find a large variety of substrates in addition to soil.

According to the origin, the substrates can be classified as organic or inorganic. Inorganic ones include sand (Muchjajib et al., 2015), perlite (Işık et al., 2020), vermiculite (Bulgari et al., 2021), rockwool (Maluin et al., 2021) and polyethylene-terephthalate foams (Kyriacou et al., 2016). Among the organic, it is worth mentioning peat moss (Murphy and Pill, 2010), compost (Carolyn F. Weber, 2017), vermicompost (Muchjajib et al., 2015), coconut fiber (Kyriacou et al., 2020), jute (Bulgari et al., 2021), sugarcane filter cake (Muchjajib et al., 2015), as well as diverse by-products of local industries (D'Imperio et al., 2021). In particular, for microgreens cultivation, the use of growing pads has become widespread (Carolyn F Weber, 2017). According to the material they are made of, it is possible to distinguish between synthetic fibers as biopolymers or polyethylene-terephthalate, and natural fibers, such as coconut, kenaf, wool, cotton, jute (Di Gioia et al., 2017) or hemp(Łaźny et al., 2021). The current trend is both peat moss, alone or mixed with perlite or vermiculite, and coconut fiber. These most widely used substrates are described as follows.

- Peat moss: is a fibrous media composed of plant material (typically Sphagnum moss) that has been partially decomposed under anaerobic, waterlogged conditions (Barrett et al., 2016). It is the most chosen media because of its excellent performance in terms of water retention and aeration (BeltranoGimenez, 2015) but has the disadvantage, from an environmental point of view, being a non-renewable resource.
- Vermiculite and perlite: both of them are mineral materials industrially processed, from mica and volcanic rocks respectively subjected to high temperatures (BeltranoGimenez, 2015). They are generally used mixed with some other organic substrates, to provide porosity, aeration, water retention, and in vermiculite case also cation exchange capacity.
- Coconut fiber or coir: is a waste product from the coconut (*Cocos nucifera*) industry (Arenas et al., 2002). It provides a favorable balance of air and water to plants’ roots (Barrett et al., 2016). In recent years, it has become an alternative to the use of peat, due to its similar characteristics, and the advantage of being a more environmentally sustainable option. However, as coconut fiber can present high levels of salinity, it is necessary to wash it before using it as a substrate.

Scientific information about the impact of organic, synthetic, and novel by-product substrates on phytochemical profiles is still scarce. Reports on this matter show just a glimpse of the possible influence of the different substrates on phytochemical biosynthesis (Kyriacou et al., 2020).

**Fig. 2:**
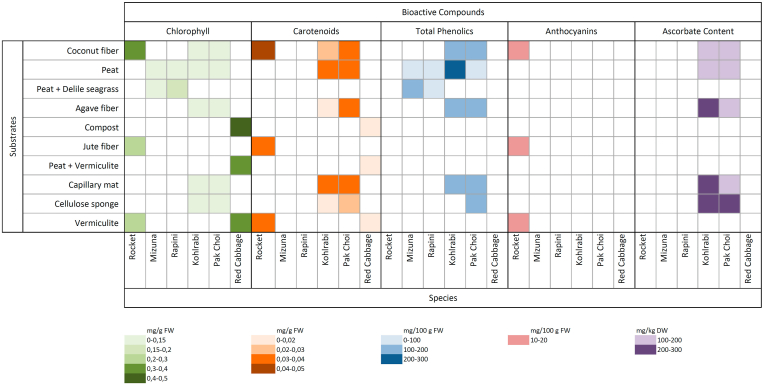
Color map of the influence of substrates on the phytochemical content of species of microgreens. FW: Fresh weight. DW: Dry weight. (For interpretation of the references to color in this figure legend, the reader is referred to the Web version of this article.)

Table 2 and Fig. 2 resume recent reports on the influence of substrates on the phytochemical quality of microgreens. In this sense, each vegetable species shows different results regarding the substrates considered. When using coconut fiber for rocket (*Eruca sativa* Mill.) (Bulgari*et al.*, 2021) or pakchoi (*Brassica rapa* L. *subsp. chinensis*) (Kyriacou et al., 2020) increments in chlorophyll, carotenoids and total phenolic content were reported. In contrast, in kohlrabi (*Brassica oleracea* L. var. gongylodes), increments occurred when peat was used. As a strategy to reuse plant residues, D'Imperio et al. (D'Imperio et al., 2021) reported that adding Delile seagrass residues (*Posidonia oceanica* L.) to peat contributed to enhance the contents of chlorophyll in rapini (*Brassica rapa* L. *rapini group*) and total phenolics in mizuna (*Brassica rapa* L. *mizuna group*). In this sense, also the use of compost, as a substrate based on organic wastes, has shown, in a different work, for red cabbage (*Brassica oleracea* var. *capitata f. rubra*) the same effect on chlorophyll content (Wieth et al., 2019). Synthetic alternatives were tested by Kyriacou (Kyriacou et al., 2020) in kohlrabi (*Brassica oleracea* L. *var. gongylodes*) and pakchoi (*Brassica rapa* L. *subsp. Chinensis*). They reported diverse responses depending on the species, but for total ascorbate content, it is possible to observe higher levels in general when using synthetic substrates as capillary mats or cellulose sponges.

**Table 2:** Articles on substrates and phytochemical profiles in Brassicaceae microgreens.

Treatments	Species/Cultivars	Bioactive compounds	References
Coconut fiber	Rocket *(Eruca sativa Mill.)*	Total Chlorophyll	Bulgari et al. (2021)
Jute fiber		Total Phenolic content	
Vermiculite		Carotenoids	
		Anthocyanins	
Peat	Mizuna *(Brassica rapa* L. *Mizuna group)*	Total Chlorophyll	D'Imperio et al. (2021)
Peat mixed with *Posidonia oceanica*	Rapini *(Brassica rapa* L. *Rapini group)*	Total Phenolic content	
		Carotenoids	
Agave fiber	Kohlrabi *(Brassica oleracea* L. *var. gongylodes)*	Total Chlorophyll	Kyriacou et al. (2020)
Capillary mat	Pakchoi *(Brassica rapa* L. *subsp. chinensis)*	Lutein	
Cellulose sponge		β-carotene	
Coconut fiber		Total Ascorbic acid	
Peat moss		Total Phenolic content	
Vermiculite (CSC®)	Red cabbage (*Brassica oleracea* var. *capitata f. rubra*)	Total Chlorophyll	(Wieth et al., 2019)
Peat + Organic waste of grape and rice industries (Beifiur® S10)		Carotenoids	
Peat + Vermiculite + Calcareous			
(Carolina Soil® seedling)			
Peat + organic waste of rice industry + vermiculite + perlite (Carolina Soil® organic)

Fiber substrates, such as coconut fiber or its mixtures with organic waste, in terms of the effect on phytochemical profiles, have shown a good general performance, which makes them a valid alternative to enhance bioactive compounds of interest. In addition, fiber substrates are suitable to replace peat and are more environmentally sustainable. In this sense, synthetic substrates also represent competitive alternatives (Kyriacou et al., 2020), although, in this type of material, it is essential to consider environmentally responsible disposal at the end of production (Bulgari et al., 2021). In any case, it is convenient to take into account the notable influence of the species in the phytochemical analysis regarding substrate election (Kyriacou et al., 2020).

### 2.3 Light conditions

Light is one of the most determinant crop factors, especially in indoor farming where artificial lighting provides radiation for photosynthesis and light signaling. Fluorescent tubes, or high-intensity tubes, such as high-pressure sodium (HPS), are artificial light sources that have been widely used (Ouzounis et al., 2015). Nowadays, the use of horticultural LED (light-emitting diode) has replaced the old light sources due to their potential for high energy efficiency and durability, long life, and low heat emissions directed towards the crop (Mitchell and Stutte, 2015).

Researchers from all over the world analyze different alternatives to optimize lighting parameters. Intensity and quality of light are important factors that affect the development of plants and regulate their behavior (Whitelam and Halliday, 2007). Plants not only detect light in its quantity (fluency rate), but also in terms of its quality (wavelength, i.e. color), direction, and duration (photoperiod) (Christie et al., 1999; Brazaityte et al., 2015).

**Fig. 3:**
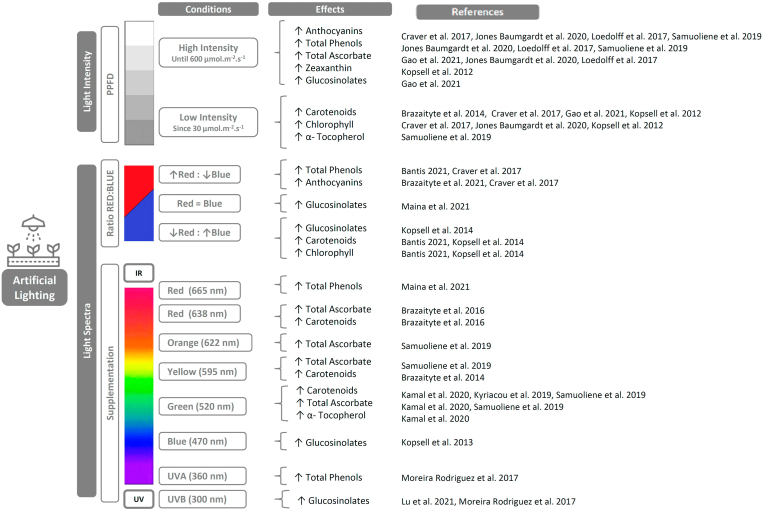
Research on artificial lighting conditions and reported effects on phytochemical profile in *Brassicaceae* microgreens. PPFD: Photosynthetic Photon Flux Density.

Particularly for microgreens, which are well suited to vertical cultivation in controlled environments, managing lighting conditions is essential to optimize yields, and can also be used as a tool to model their phytochemical profile (Jones-baumgardt et al., 2019; Ying et al., 2021; Ebert, 2022). In this regard, research on artificial lighting has focused on two main aspects: the effect of different light intensities, and the effect of supplementation with different light spectra and the ratios between them. The reported results are presented in Fig. 3, and certain trends can be observed for particular conditions.

When testing different light intensities, the photosynthetic photon flux density (PPFD) is taken as a measure. The ranges that are used go from low light intensities of 30 μmol m^−2^.s^−1^ to high intensities that go up to 600 μmol m^−2^.s^−1^. Although it is not appropriate to generalize about the effects of each treatment, because in many cases the responses are species-specific, some coincidences in the reported results allow certain trends to be inferred. Increasing irradiance intensities could induce a mild photostress (Verlinden, 2020), and lead to lower levels of chlorophyll in kohlrabi, mustard, mizuna, cabbage, and arugula (Kopsell et al., 2012; Craver et al., 2017; Jones-Baumgardt et al., 2021). Similar responses were reported for carotenoids in mustard, red pakchoi, tatsoi, mizuna, and broccoli (Kopsell et al., 2012; Brazaityte et al., 2015; Craver et al., 2017; Gao et al., 2021). On the other hand, zeaxanthin, had the opposite response, increasing with a high-intensity light pre-harvest treatment (Kopsell et al., 2012). Besides, other research reported no changes in carotenoid levels with variations in light intensity for cabbage, mustard, arugula, and kale (Jones-Baumgardt et al., 2021). In the case of compounds with antioxidant properties, such as anthocyanins, total ascorbate and total phenols, high light stress by inducing reactive oxygen species (ROS) would generate the production of antioxidant compounds in order to scavenge ROS (Loedolff et al., 2017). This would explain that increasing light intensities enhanced the content of anthocyanins in kohlrabi, kale, cabbage, arugula, mustard, wild rocket, red pakchoi and tatsoi (Samuoliene et al., 2013; Craver et al., 2017; Loedolff et al., 2017; Jones-Baumgardt et al., 2021); of total phenols in kale, mustard, cabbage, arugula, wild rocket, kohlrabi, red pakchoi and tatsoi (Samuoliene et al., 2013; Loedolff et al., 2017; Jones-Baumgardt et al., 2021), and total ascorbate in broccoli, kale, mustard, cabbage, arugula and wild rocket (Loedolff et al., 2017; Gao et al., 2021; Jones-Baumgardt et al., 2021). Regarding the effect of PPFD on glucosinolates content, Gao et al. (2021) studied the effect of low light intensities on broccoli and reported the highest contents of glucobrassicin and glucoraphanin when using higher levels, specifically 70 μmol m^−2^.s^−1^.

In addition to light intensity, variations in light quality, either by supplementation or by modifying the proportion of different wavelengths, may also have effects on some bioactive compounds in *Brassicaceae* microgreens. Numerous investigations have studied the proportions of red and blue light. According to the results when increased the proportion of red light, total phenols content was enhanced in radish (Bantis, 2021), same occurred in kohlrabi with a combination of Red: Far Red: Blue light of 84:7:9 (Craver et al., 2017). Similar effects are shown for anthocyanins in mustard, whose levels decrease as the proportion of blue light increases (Brazaitytė et al., 2021). However, responses observed are species-specific; therefore the potential use of this tool as a strategy to increase this type of compound should be evaluated in particular for each species. Variation in the red/blue ratio also influences glucosinolates levels. For Ethiopian mustard, supplementation of fluorescent light with blue plus red light at a 1:1 ratio 7 days before harvest promoted an increase in the level of glucobrassicin (Maina et al., 2021). In another study, for broccoli microgreens, the highest level of glucosinolates was reported when 20% blue light and 80% red light treatment was used, this is explained by the authors by the stimulation produced by exposure to blue light on the biosynthesis of primary and secondary metabolites (Kopsell et al., 2014). Finally, regarding the use of supplementation with certain wavelengths, numerous investigations have studied different colors of supplemental light on microgreens crops. Starting with UV, two different studies on broccoli microgreens agree that the application of supplemental UVB radiation enhances glucosinolates levels, apparently caused by a positive regulation in the genes related to the biosynthesis pathways of secondary metabolites that respond as a defense mechanism of the plant to the attack of pathogens, injuries or stress and lead to the production of glucosinolates. Pre-harvest light supplementation for 2 h/day with UVB (312 nm) increased glucoraphanin, glucoerucin, and total aliphatic glucosinolates, and the association with the application of CaCl_2_ is proposed as a tool to prolong postharvest quality (Lu et al., 2021). This response agrees with results reported by Moreira-Rodriguez et al. (Moreira-Rodríguez et al., 2017) who observed the highest concentration of glucosinolates when applying pre-harvest high-intensity UVB radiation of spectral range between 280 and 320 nm, which increased the levels of 4-methoxy-glucobrassicin, glucobrassicin, and glucoraphanin. In addition, the same study showed higher levels of phenols when applying low-intensity UVA radiation of a spectral range between 320 and 400 nm. Supplementation with blue light (470 nm) for 5 days before harvest resulted in higher levels of glucosinolates for broccoli compared to a combined red:blue light treatment. Increases in aromatic and aliphatic glucosinolates, as glucoraphenin, epiprogoitrin, and gluconasturtiin stand out, while indolic glucosinolates were not affected. The authors suggest that blue light may have a differential impact depending on the amino acids involved in glucosinolate biosynthesis. They observed a positive influence of blue light on the biosynthesis of aliphatic and aromatic glucosinolates, but no effect on the biosynthesis of indole glucosinolates, which are the only ones derived from the amino acid tryptophan (Kopsell and Sams, 2013). Regarding green light supplementation (520 nm), under conditions of a combination of 10% green LED light with 70% red and 20% blue light the vitamins concentrations, in particular β-carotene, α-tocopherol, and ascorbic acid were enhanced in purple kohlrabi, red cabbage, broccoli, kale, red komatsuna, tatsoi and green cabbage microgreens (Kamal et al., 2020). Green light also increased levels of ascorbic acid in broccoli and carotenoids in mizuna according to a different study (Samuoliene, 2019). With the use of supplemental yellow light (595 nm), increments in carotenoids were achieved in tatsoi (Brazaityte et al., 2015). In kohlrabi, yellow light enhanced the levels of ascorbic acid, while for mizuna the same effect was achieved with orange light (595 nm) (Samuoliene, 2019). Finally, concerning the effect of supplemental red light, in mustard, red pakchoi, and tatsoi, the use of 638 nm red light for 3 days before harvest, due to the effect of photo stress, generated increments in the levels of ascorbic acid and β-carotene (Brazaitytė et al., 2016). A different study, in ethiopian mustard reports increments of total phenols when using 660 nm red light supplementation before harvest (Maina et al., 2021).

In summary, the aforementioned results indicate that lighting treatments intervene significantly in the growth and phytochemical accumulation of *Brassicaceae* microgreens; although in some cases the exact mechanisms are still unknown. Even though this review allows certain trends to be inferred from specific lighting treatments to trigger increases in bioactive compounds of interest, it must be taken into account that a large part of the results reported are species-specific and the effects are often correlated with other cultivation variables or even elicitors. In this sense, it would be inappropriate to give specific recommendations, although identifying potential lighting treatments to improve the phytochemical profile can be useful as a starting point for the evaluation of specific treatments.

### 2.4 Fertilization

For optimal plant growth, it is essential to meet their nutritional requirements. Fertilization is a fundamental practice for crop management, as it provides the necessary macro and micronutrients. It should be clarified that these requirements are specific by species, phenological stage, and vary according to the organ of the plant that is harvested.

With the hydroponic cultivation technique, Hoagland and Arnon developed a universal nutrient solution in 1950 (Hoagland and Arnon, 1950). Even though it is not ideal for meeting the specific requirements of each species, it is suitable for many cultivated species and is widely used for research.

When growing microgreens, fertilization is often skipped, due to the short cultivation cycles and the fact that seeds have the necessary reserve nutrients for germination and initial growth (T. T. Li et al., 2021). Species-specific nutritional requirements for microgreens have not yet been established either (Bulgari et al., 2017; Li et al., 2021).

In *Brassicaceae* species, nutritional management also has effects on secondary metabolism. Changes in the balance of N and S may affect the biosynthesis of different bioactive compounds as glucosinolates or phenolics (Francisco et al., 2017). For this reason, the possibility of modulating the phytochemical composition of *Brassicaceae* microgreens in response to specific fertilization is of great interest. Despite the latter, research and recommendations of specific cultural fertilization practices with the aim of improving the phytochemical profile in microgreens are still scarce (Li et al., 2021).

The studies carried out to date that concern us, mostly focus on the suppression of nutrients, and their possible effect on the plant to trigger a stress response, thus generating a higher content of secondary metabolites. The regulation of nitrate content is also studied. The responses in phenolic content, chlorophyll and vitamins, in turn associated with morphological characters and yield, are evaluated.

For this aspect, we considered studies that were carried out under different conditions using the universal nutritional solution with various strengths. The general trends are summarized in Table 3. We can observe, increases in the content of total ascorbic acid and anthocyanins when the nutrient supply is reduced in wild rocket, green cabbage, radish, and garden cress, but not so in Brussels sprouts (El-Nakhel et al., 2021; Keutgen et al., 2021). Nutrient deprivation caused decreases in chlorophyll and carotenoids contents for radish, garden cress and mustard (Keutgen et al., 2021; Kyriacou et al., 2021), whereas the effects on carotenoids for wild rocket were different depending on the experiment (El-Nakhel et al., 2021; Kyriacou et al., 2021). Furthermore, as reported by Palmitessa et al. changes in the NH_4_:NO_3_ molar ratio affected the level of carotenoids but not that of tocopherols (Palmitessa et al., 2020). According to reported results in the analyzed studies, the content of total phenols would not improve with nutritional stress. Some studies indicate that the levels remained unchanged in garden cress and mustard (Keutgen et al., 2021; Kyriacou et al., 2021), and even in certain cases, like wild rocket and radish cotyledons, decreases were observed in the level of total phenols (El-Nakhel et al., 2021; Keutgen et al., 2021; Kyriacou et al., 2021). Finally, the research carried out on the effect of calcium applications in broccoli microgreens showed that the increase in shelf life would be explained by the increase in the content of glucosinolates (Sun et al., 2015; Lu et al., 2018).

**Table 3:** Research on fertilization and phytochemical profile in *Brassicaceae* microgreens.

Treatments	Species/Cultivars	Effects on bioactive compounds	References
a) ½ strength Hoagland solution in microgreens	Rocket (*Eruca vesicaria (L.) Cav. subsp. sativa (Mill.) Thell*.)	Nutritional requirements are lower in microgreens compared to their mature counterpart.	Bulgari et al. (2017)
b) ½ strength Hoagland solution in baby leaf		Chlorophylls, carotenoids, phenols, and anthocyanins contents were lower in microgreens.	
c) ½ strength Hoagland solution in adult stages			
a) ¼ strength Hoagland solution	Wild rocket Napoli (*Diplotaxis tenuifolia*)	Without fertilization:	El-Nakhel et al. (2021)
b) Distilled water	Green Brussels sprouts Mezzo Nano (*Brassica oleracea* var. *gemmifera*)	Wild rocket:	
	Green cabbage Copenhagen (*Brassica* L. *oleracea var. capitata*)	↑ total ascorbic acid, anthocyanins, lutein and β-carotene	
		↓ yield, ↓ total phenolic acids	
		Brussel sprouts:	
		No differences	
		Cabbage:	
		↑ total ascorbic acid, anthocyanins	
a) 100% Hoagland solution	Radish (*Raphanus sativus* L.)	With decreasing nutrient supplementation in:	Keutgen et al. (2021)
b) 50% Hoagland solution	Garden cress (*Lepidium sativum* L.)	Radish cotyledons:	
c) 25% Hoagland solution		↓ carotenoids, chlorophyll, total phenols	
d) Tap water		Radish stems:	
e) Demineralized tap water		↑ anthocyanins, total phenols	
		Garden cress:	
		↑ anthocyanins	
		↓ carotenoids, chlorophyll	
		= total phenols	
Nutrient deprivation before harvest (DBH):	Mustard cv. Osaka purple (*Brassica juncea (L.) Czern*)	With nutrient deprivation before harvest in:	Kyriacou et al. (2021)
a) 0 days DBH	Rocket cv. Wild Rocket, Napoli (*Diplotaxis tenuifolia)*	Mustard:	
b) 6 days DBH		= total phenolic content	
c) 12 days DBH		↓ carotenoids	
		Rocket:	
		↓ total phenolic content, carotenoids	
1.a) ½ strength Hoagland solution	Broccoli raab var. ‘Cima di rapa novantina’ (*Brassica rapa* L. *subsp. sylvestris L. Janch. var esculenta Hort*)	Decreasing strengths of nutrient solutions:	Palmitessa et al. (2020)
1.b) ¼ strength Hoagland solution	Broccoli cv ‘Broccolo natalino’ (*Brassica oleracea* L. *var. itálica*)	= yield (except 1/8 strength in broccoli raab)	
1.c) ⅛ strength Hoagland solution	Cauliflower cv ‘Cavolfiore violetto’ (*Brassica oleracea* L*.var. botrytis*)	↓ seedling height	
2.a) 5:95 NH4:NO3 molar ratio ½ strength Hoagland solution		Different NH4:NO3 molar ratios:	
2.b) 15:85 NH4:NO3 molar ratio ½ strength Hoagland solution		= α-tocopherol	
2.c) 25:75 NH4:NO3 molar ratio ½ strength Hoagland solution		↑ β-carotene with 25:75 NH4:NO3	
a) Calcium chloride (CaCl2) 10 mM pre-harvest	Broccoli (*Brassica oleracea* L. var. italica)	Pre-harvest calcium applications:	Sun et al. (2015)
b) Water		↑ glucosinolates level (aliphatics and indolics)	Lu et al. (2018)

Information is still insufficient to establish conclusive trends. There are differences in the responses observed in the levels of phytochemicals to nutritional treatments according to the species, stage of development and organ of the plant studied. Although in this section we focus on the nutritional management of the crop as a possible instrument to modulate the phytochemical profile, it is important in turn, to take into account the associated effects on yield and quality. Determining the actual requirements per species under standardized conditions would be a helpful first step to analyze whether fertilization is justified in productive terms and how to manage it to improve the profile of bioactive compounds.

## 3 Health benefits of microgreens

The relationship between food and health is a well-known phenomenon. Long evidence has proved that fruits and vegetables (F&V) are vital for a healthy diet. Epidemiological evidence has shown that F&V consumption helps mitigate the incidence of prevalent chronic diseases, such as diabetes, obesity and hypertension, which year after year generate deterioration in people's life quality (Vattem and Maitin, 2016). Microgreens, in this context, stand out as novel sources of physiologically active substances with highly-value effects (Jambor*et al.*, 2022).

Before delving into the bioactivities associated with microgreens, it is also important to consider phytochemicals' bioavailability. The presence of these compounds in food matrices is not enough to guarantee that biological properties would be verified (Ramirez et al., 2017). Bioactive compounds must be able to overcome several biological processes and barriers in order to reach the target sites where they would exert their biological response. Therefore, bioaccessibility and human absorption information of dietary intake phytochemicals, such as polyphenols, glucosinolates and/or isothiocyanates, are key factors in assessing their significance in human health (Tomas et al., 2021). Several studies carried out on *Brassicaceae* sprouts and microgreens have shown glucosinolates, isothiocyanates and phenolic compounds remain bioaccessible even after *in vitro* gastrointestinal digestion (Sun et al., 2015; Beatriz de la Fuente et al., 2019; Abellán et al., 2021a, 2021b; Tomas et al., 2021). More studies focused on the intestinal absorption, metabolism and blood stability of microgreens’ phytochemicals are still needed. Nevertheless, there is information about isothiocyanates absorption by enterocytes or colon epithelial cells, and then, free isothiocyanates along with their conjugates, are absorbed by peripheral organs, accumulating, lastly, in cells by reacting with thiol groups of glutathione and proteins (Oliviero et al., 2018). Phenolic compounds, on the other side, do not cross the intestinal barrier so easily. Numerous authors have stated that polyphenols' bioavailability is not high, because of their poor absorption, chemical instability, excessive metabolism and/or intestinal microbial transformation. Despite this, many phenolic compounds show biological responses even at low plasma concentrations (Abourashed, 2013). The food matrix influence should be considered in this process (Parada and Aguilera, 2007; Torres-Palazzolo et al., 2021), so specific studies addressed microgreens’ phytochemicals, taking into account the release, transformation, and subsequent absorption of the active compounds in the digestive tract are important factors to fully comprehend the phytochemicals bioavailability.

Now, in terms of microgreens biological effects, as discussed in previous sections, the broad spectrum of compounds present in *Brassicaceae* microgreens has been proposed as the responsible for the bioactive attributes of these plant matrices (Choe et al., 2018; Zhang et al., 2021; Sharma et al., 2022). Most of these properties correspond to preliminary studies on the potential biological mechanisms affected by the phytochemicals present in microgreens, hence, indirectly implying the prevention of some chronic diseases (Teng et al., 2021). Only a few studies approaching direct confirmation of the biological properties based on *in vitro* cell assays and animal models related to microgreens effects. Table 4 resumes these *in vitro/in vivo* studies carried out on *Brassicaceae* microgreens. In these specific investigations, phenolic compounds and/or ITCs/indoles have been proposed as the moderating agents of anti-inflammatory activities and immunoprotective (promoted by antioxidant effects, reduction of liver cytokines, and inhibition of the LPS-induced activation of the NF-κB signaling pathway and the secretions of inflammatory proteins)(Huang et al., 2016; Subedi et al., 2019; Wojdyło et al., 2020; Jambor et al., 2022); anticancer properties (antiproliferative effect on colon cancer –based on a Caco-2 cells model (Fuente et al., 2020), anti-proliferative and pro-apoptotic effects in 3D cell cultures (Ewin sarcoma)(Truzzi*et al.*, 2021), anti-proliferative effects on human prostate carcinoma (Drozdowska et al., 2021), and proliferative inhibition and anti-migrating effects on breast and liver cancer cells (Saengha et al., 2021); cardiovascular disease (CVD) prevention (by modulation of lipidic uptake and oxidative stress metabolism) (Huang et al., 2016; X. T. Li et al., 2021; Ma et al., 2022); anti-diabetic effects (by inhibition of α-amylase, and/or affecting glucose uptake) (Wadhawan et al., 2018; Aly et al., 2020; Wojdyło et al., 2020; Ma et al., 2022); and gut-microbiota modulation(protection against gut inflammation by enhancing the production short fatty acids, and improving gut bacterial diversity)(Wojdyło et al., 2020; Ma et al., 2022).

**Table 4:** Functional properties of different types of microgreens and the bioactive compounds to which bioactivity is attributed.

Functional property	Species/Cultivars	Bioactive compound which bioactivity is attributed	References
Anti-diabetic and anticholinergic activity	Radish *(Raphanus sativus)*, amaranths *(Amaranthus)*, kale *(Brassica oleracea)*	Carotenoids, chlorophylls and organic acids	Wojdyło et al. (2020)
Lower circulating LDL, reductions in hepatic cholesterol ester	Red cabbage*(Brassica*		Huang et al. (2016)
Reduction in triacylglycerol levels and expression of inflammatory cytokines	*oleracea L. var. capitata)*		
Anti-neuroinflammatory and neuroprotective activities	Broccoli sprouts*(*Brassica oleracea* L. *var. itálica)*	Sulforaphane	Subedi et al. (2019)
Potential to alleviate hyperglycemia	Radish *(Raphanus sativus)*		Aly et al. (2020)
Antioxidant effects in cases of diabetic state or for prevention of this disease.			
Properties anticancer and DDPP antioxidant activity	Mustard green *(Brassica juncea)*	Isothiocyanates and phenolic compounds	Saengha et al. (2021)
Reduction of cell proliferation of Ewin sarcoma (3D cell cultures)	Red Rambo radish *(Raphanus sativus),* rocket *(Eruca vesicaria* subsp. *sativa)*	Polyphenols	Truzzi et al. (2021)
Antiproliferative on both RD-ES and A673 sarcoma spheroids	Green pea *(Pisum sativum)*		
Potential anti-tumor effect	Red rambo radish *(Raphanus sativus)*		
Correction of glycemic dysregulation, weight reduction in type 2 diabetes	Broccoli (*Brassica oleracea* L. *var. italica)*		Ma et al. (2022)
Improvement of the microbial structure of the intestine in type 2 diabetes			
Reduction of white adipose tissue mass, body weight and size of adipocytes, improvement of glucose tolerance, reduction of insulin level and resistance	Broccoli (*Brassica oleracea* L. *var. italica)*		(X. X. Li et al., 2021)
Antiproliferative effect in the colon cancer Caco-2 cells	Broccoli (*Brassica oleracea* L. var. *italica)*, kale (*Brassica oleracea* var. *sabellica* L.), mustard (*Brassica juncea* (L.) Czern.), and radish (*Raphanus sativus*L.)	Soluble phenolic compounds, isothiocyanates and ascorbic acid	Fuente et al. (2020)

From this information, one can argue that cruciferous microgreens possess a unique mixture of bioactive compounds that consequently evidences a spectrum of biological activities probably caused by additive/synergistic mechanisms and metabolic pathways. From here arises the importance to study each product particularly. To deepen more detailed scientific health-claims regards these matrices, comprehensive studies including *in vivo* and epidemiological data should be addressed.

## 4 Conclusions

Microgreens have emerged as a novel alternative for healthy and sustainable eating, and the Brassicaceae family is highlighted due to the variety of health benefits reported.

In the present work, we carried out an analysis of the impact of crop factors involved in production on the phytochemical profiles of different species. Thereby, the reviewing of certain agricultural practices concerning types of substrates, artificial lighting, and the use of fertilizers, allowed an insight into potential crop management strategies to improve the richness in bioactive compounds and obtain functional foods.

Nevertheless, it is important to note that results are mostly species-specific. Besides, when analyzing the treatments’ effects, it would be convenient to do it concerning the particular cultivation conditions of each experiment due to their possible synergistic effects. In general, we can conclude that fiber substrates could be considered a valid and sustainable alternative for enhancing bioactive compounds of interest. Artificial lighting treatments intervene in growth and phytochemical accumulation, although further research is still needed on the exact mechanisms involved. Lastly, to modulate bioactive compound profiles in response to fertilization would be possible; however, research and recommendations on actual requirements and nutrition management per species to improve phytochemical contents are still scarce.

Although production and research in this area have become a trending topic in the last 10 years, several aspects such as bioactivity and bioavailability remain to be explored in depth.

## Funding

This research was supported by SIIP 10.13039/501100005961Universidad Nacional de Cuyo (Project SIIP 06/A007T1.) CONICET (PIP11220200101692CO) and MINCyT-PICT 2019-03278, Argentina.

## CRediT authorship contribution statement

Florencia P. Alloggia: Conceptualization, Writing - Review & Editing, Visualization. Roberto F. Bafumo: Writing - Review & Editing. Daniela A. Ramirez: Writing - Review & Editing. Marcos A. Maza: Writing - Review &Editing. Alejandra B. Camargo: Conceptualization, Writing, Supervision.

## Declaration of competing interest

The authors declare the following financial interests/personal relationships which may be considered as potential competing interests: Alejandra B. Camargo reports financial support was provided by MINCYT. Alejandra B. Camargo reports a relationship with CONICET Mendoza that includes: employment.
